# Surgical Outcome Risk Tool (SORT) to predict 30-day postoperative mortality in a mixed surgical population in Swedish tertiary hospitals

**DOI:** 10.1093/bjs/znad039

**Published:** 2023-03-10

**Authors:** Egidijus Semenas, Johan Helleberg, Erzsébet Bartha, Sigridur Kalman, Manne Holm

**Affiliations:** Department of Surgical Sciences, Anaesthesiology and Intensive Care, Akademiska Sjukhuset, Uppsala, Sweden; Department of Clinical Science, Intervention, and Technology, CLINTEC, Karolinska Institutet, Stockholm, Sweden; Perioperative Medicine and Intensive Care, B31, Karolinska University Hospital, Huddinge, Sweden; Department of Clinical Science, Intervention, and Technology, CLINTEC, Karolinska Institutet, Stockholm, Sweden; Perioperative Medicine and Intensive Care, B31, Karolinska University Hospital, Huddinge, Sweden; Department of Clinical Science, Intervention, and Technology, CLINTEC, Karolinska Institutet, Stockholm, Sweden; Perioperative Medicine and Intensive Care, B31, Karolinska University Hospital, Huddinge, Sweden; Department of Clinical Science, Intervention, and Technology, CLINTEC, Karolinska Institutet, Stockholm, Sweden; Perioperative Medicine and Intensive Care, B31, Karolinska University Hospital, Huddinge, Sweden

## Abstract

**Background:**

The Surgical Outcome Risk Tool (SORT) was derived and validated in the UK to improve preoperative prediction of postoperative risk. The aim of this study was to validate the SORT in a European mixed-case surgical population outside of the UK.

**Methods:**

The study included patients aged at least 18 years with ASA Physical Status (ASA-PS) grades I–V who underwent non-cardiac surgery at four tertiary hospitals in Sweden between November 2015 and February 2016. Exclusion criteria were surgery under local anaesthesia and missing data on the SORT predictors (ASA-PS, surgical urgency, high-risk surgery, surgical severity, malignancy, age over 65 years). The outcome was 30-day mortality. Discrimination and calibration of the SORT were assessed using area under the receiver operating curve (AUROC) statistics and calibration plots. A sensitivity analysis was done in a high-risk subgroup (ASA-PS III or higher; surgical complexity major to Xmajor according to the SORT; gastrointestinal, orthopaedic, urogenital/obstetric surgery; and age at least 18 years).

**Results:**

The validation cohort included 17 965 patients; median age was 58 (i.q.r. 40–70) years, 43.2 per cent were men, and the mortality rate at 30 days was 1.6 per cent. The SORT had excellent discrimination, with an AUROC of 0.91 (95 per cent c.i. 0.89 to 0.92), and good calibration. The high-risk subgroup (1807 patients) had a 30-day mortality rate of 5.6 per cent; in the sensitivity analysis, the SORT had good discrimination, with an AUROC of 0.79 (0.74 to 0.83), and calibration remained good.

**Conclusion:**

The estimates of the original the SORT for prediction of 30-day mortality were valid and reliable in a mixed-case surgical population in a non-UK European setting.

## Teaser

The Surgical Outcome Risk Tool (SORT) was developed for prediction of postoperative mortality in a prospective UK development study, but a later validation study from New Zealand showed underestimation of risk and a need for recalibration. The present study included a mixed surgical cohort of 17 965 patients, with many demographical similarities to the original SORT development cohort, and aimed to validate SORT for prediction of postoperative mortality. The original SORT was found to be valid and reliable in this mixed-case European non-UK surgical population hospitals for prediction of 30-day mortality.

## Introduction

Timely access to safe and affordable surgical and anaesthesia care is essential for the full attainment of local and global healthcare goals^1^. Guidelines^2^ specifically recommend adequate preoperative risk assessment to assist decision-making for both clinicians and patients regarding the appropriateness of surgery, surgical approach, monitoring, and postoperative care. To assist preoperative risk assessment, the most commonly used risk classification tool for non-cardiac surgery is the around 60-year-old ASA Physical Status (ASA-PS) classification^3^. It does not, however, provide individually calculated patient risk for negative outcomes, and several risk prediction models are available for such purposes^4,5^. There is a plethora of prognostic models to predict postoperative mortality or morbidity but, to adopt any tool either for clinical use or for research, local validation is required^6^. Examples of such tools include the American College of Surgeons National Surgical Quality Improvement Program, Portsmouth version of POSSUM, and the Surgical Outcome Risk Tool (SORT)^7–9^.

Supported by a previous systematic review^4^, the authors identified the SORT as potentially suitable as it incorporates six readily available preoperative variables, and has potential for easy electronic data extraction from different national registries in Swedish healthcare settings. The SORT was derived and validated in the UK; it has shown excellent discrimination and, naturally, varying accuracy in external validation cohorts, with both underestimation and overestimation of risk in different data sets extracted from a variety of clinical settings, highlighting the need for validation studies in the actual settings in which the model may be implemented^10–12^. Previous validation studies have been done on cohorts in the UK, New Zealand (NZ), Australia, and Brazil; however, to the authors’ knowledge, no validation study in a large mixed surgical cohort has been done in a European healthcare system outside of the UK. The aim of this study was to validate the SORT for prediction of 30-day postoperative mortality in a Swedish mixed-case surgical population.

## Methods

The manuscript was prepared according to the TRIPOD statement^6^. An annotated checklist with items from the TRIPOD statement is provided in *Table S1*. The study was approved by the Regional Ethical Committee in Stockholm (IRB, reference number 2015/1128-31/4). Informed consent was waivered by the regional ethical review board in Stockholm.

### Source of data

The validation data set was extracted from the background population of a previous prospective study^13^ of postoperative outcomes in a high-risk surgical cohort of 1063 patients. The background population, according to the STROBE guideline^14^, consisted of all patients who underwent surgery at the included centres during the study interval. Inclusion was done between November 2015 and February 2016 at five study sites in four tertiary hospitals: Karolinska University Hospital Huddinge and Solna, Akademiska University Hospital in Uppsala, University Hospital in Örebro, and University Hospital in Linköping (NCT02626546). The previous prospective study was a multicentre observational closed cohort consecutive, interrupted parallel time study. It was conducted in accordance with the Handbook for Good Clinical Research Practice (WHO), in line with the principles of the Declaration of Helsinki, the Swedish Personal Data Act, and the Personal Data Ordinance. Personal data processing was authorized by the Swedish Data Protection Agency and approved by the Regional Ethical Committee, Stockholm (2015/1128-31/4).

The background population was used for this retrospective external validation study. Data collection was done in 2018 via electronic medical records of all patients who underwent surgery during the above recruitment period at the participating hospitals. Inclusion criteria were adult patients (aged at least 18 years), with ASA-PS grades I–V, who underwent non-cardiac surgery. Patients who underwent surgery under local anaesthesia and those with missing data on the SORT predictors (for example ASA-PS grade, type of urgency, surgical code, or age) were excluded.

### Outcome

The predicted outcome was 30-day postoperative mortality; these data were collected from the electronic online Swedish Population Register, which is continuously updated regarding all deaths in Sweden.

### Predictors

The SORT includes six predictors: ASA-PS grade, urgency of surgery (expedited, urgent, immediate), high-risk surgery (thoracic, gastrointestinal, or vascular), surgical severity (minor to major–complex), malignancy (within the last 5 years), and age over 65 years. These data were available from the operation planning software at the participating study sites. ASA-PS grade was entered by the anaesthetists into the operation planning system, and the Swedish surgical procedure codes and urgency by the surgeon in charge. Data on malignancies were extracted from the National Swedish Cancer registry by data linkage undertaken by statisticians at the Swedish National Board of Health. The criteria and definitions of the three urgency categories were not uniform at the participating centres, so all types of urgency were categorized as ‘urgent’, similar to the method applied in a previous the SORT validation study^12^. Definitions of surgical severity (minor to major–complex) were decided by one author by mapping the Swedish surgical procedures into the UK procedures and categorized by AXA PPP/Specialist Procedure Codes (AXA insurance company with Public–Private Partnership). No blinding of outcomes or predictors was performed, except during the surgical procedure code translation, where the responsible author was blinded to patient outcome. For each Swedish procedure code, the specialty was identified and the same procedure was identified in the corresponding British surgical procedures (for example oophorectomy). If it was not found, the classification of a similar code was used (for example salpingectomy). If this was unsuccessful, a free-text search was conducted to see whether the surgical procedure was available under a different specialty. In the event of any uncertainty regarding mapping a procedure, this was discussed and resolved together with two other authors. For procedures with more than one surgical code, the most complex code was used to define complexity. For procedures with codes from more than one specialty, the most complex code was used to define the surgical specialty for the patient. The detailed script for surgical code mapping is available upon request to the authors.

### Statistical analysis

During study planning, no formal sample size calculation was conducted. It was expected that the sample size would approach the size of the original the SORT derivation cohort^9^. For external validation studies, a rule of thumb with at least 100 events has been suggested previously^15,16^. In the present study, over 200 events were anticipated and so the study was presumed to have adequate power for statistical analyses of model performance. A full-case analysis was performed; no imputation or other replacement of missing data was done. The predicted risk was calculated based on the full equation of the SORT^9^: Risk score = −7.366 + ASA-PS III × 1.411 + ASA-PS IV × 2.388 + ASA-PS V × 4.081 + cancer × 0.667 + age 65–79 × 0.777 + age ≥ 80 × 1.591 + urgency expedited × 1.236 + urgency urgent × 1.657 + urgency immediate × 2.452 + high-risk specialty × 0.712 + severity Xmajor complex × 0.381.

The SORT performance was evaluated by means of discrimination and calibration analyses. For illustration and comparison, the ASA-PS grade was also analysed for discrimination. Discrimination was investigated using the C statistic, calculating the concordance index (C-index) with 95 per cent confidence interval. C-index equals the area under the receiver operating characteristic curve (AUROC). The original the SORT was validated by re-estimating the risk with 10-fold cross-validation analysis using logistic regression for 30-day mortality. This included an initial random shuffle of the data set, which was then split into 10 groups. Each subsample was retained as the validation data, with the remainder used as training data. This was then repeated 10 times, so that all observations were used for both training and validation. The results were then averaged into a single estimation. The discrimination of the re-estimated model was compared with that of the original the SORT using a test for the equality of the area under the curves based on an algorithm suggested by DeLong *et al.*^17^. The calibration analysis assessed the agreement between predicted and observed outcomes. It is reported graphically, with observed mortality plotted on the *y*-axis and the predicted risk on the *x*-axis, with the residuals (95 per cent c.i.) presented around the calibration curve. The calibration was done by tenth of the risk and was augmented by a smoothed line over the entire probability range. Calibration strength was evaluated by the calibration slope and calibration intercept. The calibration slope assesses the spread of the estimated risks with a target value of 1. Values below 1 indicate that estimated risks are too extreme, that is too high for patients at high risk and too low for those at low risk. In contrast, values above 1 suggest that risk estimates are too moderate. The calibration intercept assesses calibration as a whole with a target value of 0. Negative values suggest overestimation and positive values underestimation^18^. Categorical variables are presented as numbers with percentages. Continuous variables are presented as median (i.q.r.). Two-sided *P* < 0.050 was considered significant. Statistical analyses were performed in Stata^®^ release 15 (StataCorp, College Station, TX, USA) and surgical procedure code mapping was conducted in R (R Foundation for Statistical Computing, Vienna, Austria).

#### Sensitivity analyses

Sensitivity analyses were used to assess the effect of the collapsed predictor urgency by running the discrimination and calibration analyses for 30-day mortality with all urgencies categorized as ‘expedited’ in the SORT equation. Analyses were repeated for 30-day mortality in a restricted subgroup of high-risk patients using the inclusion criteria for the previously published high-risk cohort^13^: ASA-PS III or higher; surgical complexity major to Xmajor; gastrointestinal, orthopaedic, or urogenital/obstetric surgery; and age at least 18 years.

## Results

**Fig. 1:**
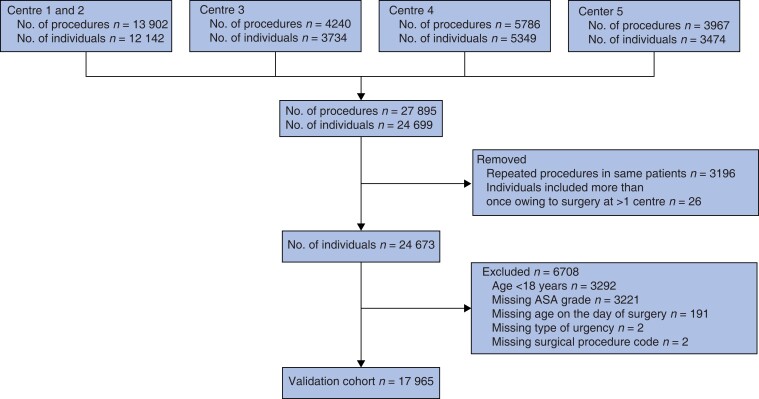
Study flow chart

During the study interval, 27 895 surgical procedures were performed. After removal of repeated procedures, individuals aged below 18 years, surgery under local anaesthesia, and those with missing data on the SORT variables for the day of surgery, the validation data set comprised 17 965 individuals (*Fig. 1*). Survival data were available for all patients; 284 (1.6 per cent) had died by 30 days after operation. Most patients underwent elective surgery, and a majority had an ASA-PS grade of I or II (*Table 1*). The most common surgical specialty was orthopaedic surgery, followed by gastrointestinal/abdominal, and urogenital. The 284 patients who had died by 30 days were older, more often men, had a higher ASA-PS grade, and more often underwent acute surgery compared with the 17 681 patients who were alive at 30 days (*Table 1*)

**Table 1:** Patient demographics

	Total cohort (*n* = 17 965)	Dead at 30 days (*n* = 284)	Alive at 30 days (*n* = 17 681)
Age at surgery (years), median (i.q.r.)	58 (40–70)	76 (68–86)	57 (40–70)
Sex ratio (M : F)	7763 : 10 202	156 : 128	7607 : 10 074
**Surgical urgency**			
ȃUrgent	4211 (23.4)	179 (63.0)	4032 (22.8)
ȃElective	13 754 (76.6)	105 (37.0)	13 649 (77.2)
**Surgical complexity**			
ȃMinor	1399 (7.8)	27 (9.5)	1372 (7.8)
ȃIntermediate	4850 (27.0)	68 (23.9)	4782 (27.0)
ȃMajor	5647 (31.4)	80 (28.2)	5567 (31.5)
ȃXmajor	2975 (16.6)	60 (21.1)	2915 (16.5)
ȃComplex	3094 (17.2)	49 (17.3)	3045 (17.2)
ȃMalignancy	4746 (26.4)	114 (40.1)	4632 (26.2)
**ASA grade**			
ȃI	5218 (29.0)	2 (0.04)	5216 (99.9)
ȃII	7622 (42.4)	31 (0.41)	7591 (99.6)
ȃIII	4486 (25.0)	139 (48.9)	4347 (24.6)
ȃIV	609 (3.4)	99 (34.9)	510 (2.9)
ȃV	30 (0.2)	13 (4.6)	17 (0.1)
**Surgical specialty**			
ȃVascular	343 (1.9)	10 (3.5)	333 (1.9)
ȃOrthopaedic	4126 (23.0)	80 (28.2)	4046 (22.9)
ȃNeurosurgery	1140 (6.3)	29 (10.2)	1111 (6.3)
ȃObstetrics	870 (4.8)	0 (0)	870 (4.9)
ȃUrogenital	2914 (16.2)	8 (2.8)	2906 (16.4)
ȃGastrointestinal/abdominal	2941 (16.4)	68 (23.9)	2873 (16.3)
ȃBreast	660 (3.7)	0 (0)	660 (3.7)
ȃThoracic	614 (1.5)	20 (7.0)	594 (3.4)
ȃOtorhinolaryngological	1291 (7.2)	15 (5.3)	1276 (7.2)
ȃOther*	2800 (15.6)	51 (18.0)	2749 (15.6)
ȃNot classifiable†	266 (1.5)	3 (1.1)	263 (1.5)
SORT-calculated mortality risk (%), median (i.q.r.)	0.3 (0.1–0.8)	5.4 (2.2–12.0)	0.3 (0.1–0.8)

Values are *n* (%) unless otherwise indicated. *Including (with 30-day mortality rate): endoscopic (623, 4.3 per cent), oral (313, 0.03 per cent), endocrine (393, 0 per cent), ophthalmological (422, 0.02 per cent), reconstructive (675, 1.2 per cent), transplant (56, 1.8 per cent), and other procedures requiring anaesthesia, for example percutaneous interventions (318, 4.1 per cent). †No corresponding UK procedure codes were found for these surgical procedures. SORT, Surgical Outcome Risk Tool.

**Fig. 2:**
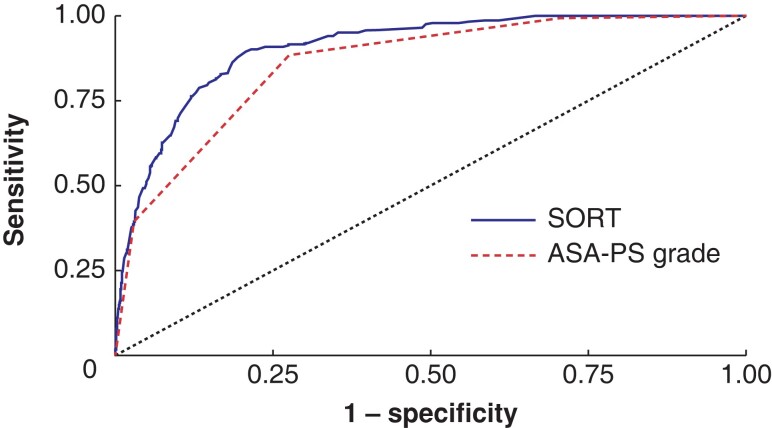
Receiver operating characteristic (ROC) curve showing discrimination of the original the SORT and that of ASA grade for 30-day mortality The area under the receiver operating curve (AUROC) of the Surgical Outcome Risk Tool (SORT) was 0.91 (95 per cent c.i. 0.89 to 0.92), which was significantly higher than that for ASA Physical Status (PS) grade only (AUROC 0.86, 0.84 to 0.88) (*P* < 0.001).

**Fig. 3:**
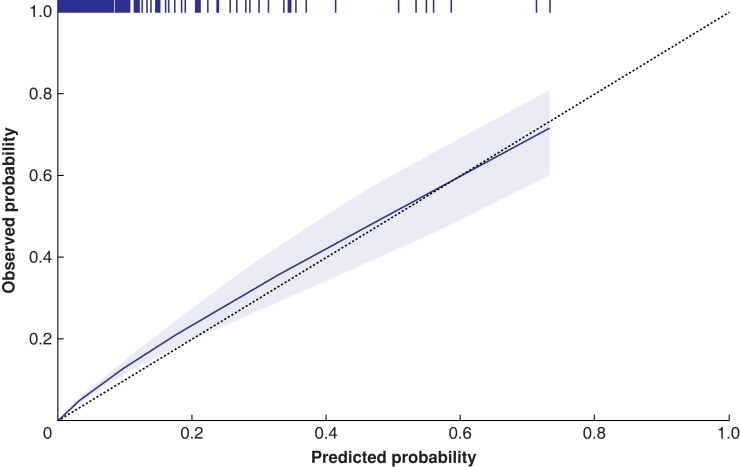
Calibration curve for mortality at 30 days for the original the SORT The slope is 1.04 (95 per cent c.i. 0.95 to 1.13) with an intercept of 0.42 (95 per cent c.i. 0.13 to 0.71). The shaded area represents the 95 per cent confidence interval. The dotted line shows optimal calibration with equal observed *versus* predicted mortality. The vertical lines at the top of the figure denotes that one or more patients have the corresponding precticted probability of death. SORT, Surgical Outcome Risk Tool.

The discrimination of the original the SORT was excellent, with an AUROC of 0.91 (95 per cent c.i. 0.89 to 0.92), which was significantly higher than that when only the ASA-PS classification was used (AUROC 0.86, 0.84 to 0.88) (*P* < 0.001) (*Fig. 2*). The SORT re-estimated via 10-fold cross-validation had an AUROC of 0.91 (0.90 to 0.93) (*Fig. S1*), which was not significantly different from that of the original the SORT (*P* = 0.156). Thus, the original the SORT was assessed in the calibration analysis. The calibration curve for 30-day mortality closely followed the optimal line, with a calibration slope of 1.04 (95 per cent c.i. 0.95 to 1.13) and an intercept of 0.42 (95 per cent c.i. 0.13 to 0.71) (*Fig. 3*).

A sensitivity analysis undertaken in the high-risk subgroup of 1807 patients with a 30-day mortality rate of 5.6 per cent (102 deaths). Demographic data are presented in *Table S2*. The SORT had good discrimination in this subgroup, with an AUROC of 0.79 (0.74 to 0.83) (*Fig. S2*), and was still well calibrated, with a slope of 0.89 (0.71 to 1.08) and an intercept of −0.04 (−0.57 to 0.49) (*Fig. S3*). In the second sensitivity analysis, using the ‘expedited’ weights in the SORT equation for all urgencies, the discrimination for 30-day mortality remained excellent, with an AUROC of 0.91 (0.89 to 0.92), and no statistical difference compared with the main analysis (*P* = 0.26). However, the reweighted model underestimated 30-day mortality, with a calibration slope of 1.12 (1.03 to 1.21) and intercept of 0.94 (0.61 to 1.28).

## Discussion

The SORT provided excellent discrimination and calibration in predicting 30-day postoperative mortality in patients who underwent surgery in four Swedish academic hospitals. In the sensitivity analysis, the model showed excellent performance in high-risk patients; the discrimination was slightly lower but calibration remained good.

The original the SORT was developed and validated in the UK using a split data set to predict 30-day mortality^9^. In more recent validation studies^10–12^, the model had excellent discrimination and a varying degree of overestimation or underestimation of the risk of death. This is attributable to the differences between case mix and healthcare settings, indicating the importance of validation. In a study from NZ, the SORT had excellent discrimination but a high degree of overestimation, indicating a need for model update and recalibration^12^. The authors added ethnicity and additional predictors for surgical specialties, and developed the NZRISK model. To facilitate the interpretation of differences in model performances between different cohorts, the prevalence of predictors in the cohorts of the original the SORT study, the present study, and the NZ study are summarized in *Table 2*. Apart from a different prevalence of malignancies, many similarities can be seen between the SORT and present Swedish data sets, which may explain the excellent model performance in the present study. The large overestimation of 30-day mortality risk in the NZ study may be related to the over-representation of minor and intermediate procedures and ASA-PS grade I or II (NZ *versus* the SORT cohort; *Table 2*). According to the authors of the NZ study^12^, the higher prevalence of high-risk surgical specialties (thoracic, vascular, neurosurgical) may have explained their need for recalibration. Indeed, neurosurgical procedures were not included in the SORT data set; however, a higher prevalence of thoracic and vascular procedures cannot be seen in the NZ cohort (*Table 2*). Nevertheless, these high-risk surgical specialties were strong predictors in the logistic regression analyses, and justified the model update by including those in the NZRISK model. However, differences in case mix cannot be the exclusive source of poor calibration in the NZ data set as those high-risk procedures were included in the present study, where excellent calibration was found.

**Table 2:** Descriptive data for different studies on the SORT

	Present study (*n* = 17 965)	Original SORT^9^ (*n* = 16 788)	NZ study^12^ (*n* = 360 140)
Age at surgery (years), mean	55.5	55.8	49.1
Sex ratio (M : F)	7763 : 10 202	7481 : 9307	166 024 : 194 116
**Surgical urgency**			
ȃElective	13 754 (76.6)	10 987 (65.4)	278 748 (77.4)
ȃExpedited	–	2136 (12.7)	–
ȃUrgent	4211 (23.4)	3424 (20.4)	81 392 (22.6)
ȃImmediate	–	241 (1.4)	–
**Severity**			
ȃMinor	1399 (7.8)	1423 (8.5)	38 895 (10.8)
ȃIntermediate	4850 (27.0)	4134 (24.6)	295 675 (82.1)
ȃMajor	5647 (31.4)	5488 (32.7)	18 007 (5.0)
ȃXmajor–complex	6069 (33.8)	5743 (34.2)	7563 (2.1)
ȃMalignancy	4746 (26.4)	1649 (9.8)	23 409 (6.5)
**ASA grade**			
ȃI	5218 (29.0)	5416 (32.3)	228 497 (63.4)
ȃII	7622 (42.4)	7585 (45.2)	117 738 (32.7)
ȃIII	4486 (25.0)	3339 (19.9)	11 586 (3.2)
ȃIV	609 (3.4)	417 (2.5)	1787 (0.5)
ȃV	30 (0.2)	31 (0.2)	532 (0.1)
**Surgical specialty**			
ȃVascular	343 (1.9)	732 (4.4)	9 220 (2.6)
ȃOrthopaedic	4126 (23.0)	5903 (35.2)	108 820 (30.2)
ȃNeurosurgery	1140 (6.3)	–	13 403 (3.7)
ȃObstetrics	870 (4.8)	–	n.a.
ȃUrogenital	2914 (16.2)	3462 (20.6)	13 962 (3.9)
ȃGastrointestinal	2941 (16.4)	2666 (15.9)	61 478 (17.1)
ȃBreast	660 (3.7)	835 (5.0)	n.a.
ȃThoracic	64 (1.5)	191 (1.1)	4113 (1.1)
ȃOtorhinolaryngological	1291 (7.2)	1257 (7.5)	n.a.
ȃOther	2800 (15.6)	1742 (10.4)	149 144 (41.4)
ȃNot classifiable	266 (1.5)	–	–
30-day mortality (%)	1.6	1.4	0.7

Values are *n* (%) unless otherwise indicated. SORT, Surgical Outcome Risk Tool; NZ, New Zealand; n.a., not available as these data in the manuscript were grouped as “Other”.

The mean(s.d.) predicted the SORT risk for the whole cohort (1.2(0.3) per cent) and the high-risk subgroup (4.6(0.6) per cent) are shown in *Table S2*. When assessing calibration in the large, that is simply comparing these numbers with the observed mortality rates of 1.6 and 5.6 per cent respectively, some underestimation is suspected^18^. This is, of course, an oversimplification as the much more stringent calibration analysis of comparing predicted *versus* observed risk across the whole risk spectra shows an almost perfect calibration curve (*Fig. 3*). However, a tendency towards underestimation may be seen in *Fig. 3* for patients at low risk (below predicted probability 0.2, supported by the intercept of 0.42 (95 per cent c.i. 0.13 to 0.71). For high-risk patients included in the sensitivity analysis, no signs of underestimation were seen (*Fig. S3*).

A problem with risk prediction studies is that models may have excellent discrimination in low-risk cohorts with few outcomes. These patients will correctly be predicted to have no event, which is in favour of the model performance, whereas there may still be a problem with poor prediction among those with predicted high risk. For this reason, a sensitivity analysis was conducted in a high-risk subgroup of 1807 patients with a more than threefold higher mortality rate than the total cohort (5.6 *versus* 1.6 per cent). As expected, the discrimination was lower (compared with that for total cohort), but remained good, with an AUROC of 0.79. The calibration was excellent and so it is concluded that the SORT remained valid in the high-risk cohort.

There are several limitations of the present work. Typically, model performance is worse in external validation cohorts^19^. The sources of model degradation in independent samples may be differences in distribution of model predictors and/or the effect of the individual predictors (coefficients) on outcome. To assess the generalizability of a model, its reproducibility (performance across different samples from the same target population) and transportability (performance across samples from independent populations) could be analysed. A full analysis of reproducibility and transportability may only be done in an independent external data set with full access to individual-patient data for both the development and validation cohorts^20^. This was not done in the present study, which may be seen as a limitation.

Full-case analyses were performed and it was assumed that missing data were missing at random. However, patients with missing ASA-PS grade were excluded (*Fig. 1*), which may have introduced bias as this group potentially includes the most severely ill patients where immediate surgery was performed and later documentation often is incomplete.

In the sensitivity analysis, the number of deaths at 30 days went down to 102 owing to exclusions, which is just at the recommended limit for external validation studies^15,16^. Thus, the analyses here may have been slightly underpowered, which is a limitation.

There is a risk of bias due to possible misclassification of three variables. The first is the ASA-PS grade, which includes subjective judgement with an inherent risk of misclassification. The classification made by the clinicians was accepted, without efforts to reclassify and assess the possible benefits of that. The second was the variable urgency of surgery. The participating study sites used non-uniform definitions for types of urgency, and in the main analysis the three urgency predictors (expedited, urgent, immediate) were collapsed into one; one single weight for urgency, the category ‘urgent’, was applied, in the SORT equation. The sensitivity analysis indicated that this variable had a considerable effect on model performance, with underestimation of mortality when the lower weight for urgency (expedited) was applied. Thus, the decision to categorize all non-elective procedures as ‘urgent’ was reasonable. The third variable was surgical severity. There is no grading system for surgical severity in Sweden and, as the mapping of the Swedish procedure codes to the AXA PPP codes was not validated, this could be a source of misclassification. Another limitation of this study is the retrospective data collection. Moreover, owing to the retrospective design of the study, it was not possible to validate the most recent the SORT^11^, to which the subjective risk estimates of clinician were added.

When assessing the performance of a risk prediction model, clinicians and patients want to know the future risk rather than the probability of an outcome^21^. For decision-making, inaccurate calibration means unreliable prediction, which impairs the utility of the model as it underestimates or overestimates the risk of death. However, the impact of additional information provided by prediction on decision-making, tailoring individualized perioperative care, and possibly improved outcome need to be addressed in future research. The lack of severity and non-uniform classification of urgency for surgical procedures in Sweden is a limiting step in the incorporation of the SORT into clinical practice in general; however, this may be overcome by development of a validated mapping tool.

## Supplementary Material

znad039_Supplementary_Data

## Data Availability

The detailed script for surgical code mapping and the study data are available upon reasonable request to the authors. No preregistration for the analysis plan of this validation study was undertaken.
