# Supplementary material for: Surgical Outcome Risk Tool (SORT) to predict 30-day postoperative mortality in a mixed surgical population in Swedish tertiary hospitals
Source: Br J Surg. 2023 Mar 10;110(5):584–90. doi: 10.1093/bjs/znad039 (PMC10364531; doi:10.1093/bjs/znad039)
Supplement: znad039_Supplementary_Data [file znad039_supplementary_data.docx]

**The Surgical Outcome Risk Tool (SORT) to predict 30-days postoperative mortality in a mixed surgical population in Swedish tertiary hospitals.**

Egidijus Semenas MD, PhD^a^; Johan Helleberg^b,c^, MD, Erzsébet Bartha^b,c^ MD, PhD, Sigridur Kalman^b,c^ MD, PhD, Manne Holm^c,d^ MD, PhD

^a^Department of Surgical Sciences, Anaesthesiology and Intensive Care, Akademiska Sjukhuset, Uppsala, Sweden

^b^Department of Clinical Science, Intervention, and Technology, CLINTEC, Karolinska Institutet, Stockholm, Sweden

^c^Perioperative Medicine and Intensive Care, B31, Karolinska University Hospital, Huddinge

^d^Department of Molecular Medicine and Surgery, Karolinska Institutet

**Corresponding author.**

Manne Holm

Perioperative Medicine and Intensive Care, B31,

Karolinska University Hospital, Huddinge

SE-141 86, Stockholm

Tel: +46-8-123 800 00.

E-mail: manne.holm@ki.se

**Supplementary Materials - Index**

| **Supplementary Figures and Tables** |  |
| --- | --- |
| Table S1 | *pag. 2* |
| Table S2 | *pag. 3* |
| Figure S1 | *pag. 4* |
| Figure S2 | *pag. 5* |
| Figure S3 | *pag. 6* |

**Supplementary Figures and Tables**

| **Table S1 TRIPOD Checklist** | | | |
| --- | --- | --- | --- |
| **Section/Topic** | **Item** | **Checklist Item** | **Page** |
| **Title and abstract** | | | |
| Title | 1 | Identify the study as developing and/or validating a multivariable prediction model, the target population, and the outcome to be predicted. | 1 |
| Abstract | 2 | Provide a summary of objectives, study design, setting, participants, sample size, predictors, outcome, statistical analysis, results, and conclusions. | 2 |
| **Introduction** | | | |
| Background and objectives | 3a | Explain the medical context (including whether diagnostic or prognostic) and rationale for developing or validating the multivariable prediction model, including references to existing models. | 3-4 |
|  | 3b | Specify the objectives, including whether the study describes the development or validation of the model or both. | 3-4 |
| **Methods** | | | |
| Source of data | 4a | Describe the study design or source of data (e.g., randomized trial, cohort, or registry data), separately for the development and validation data sets, if applicable. | 5 |
|  | 4b | Specify the key study dates, including start of accrual; end of accrual; and, if applicable, end of follow-up. | 5 |
| Participants | 5a | Specify key elements of the study setting (e.g., primary care, secondary care, general population) including number and location of centres. | 5-6 |
|  | 5b | Describe eligibility criteria for participants. | 5-6 |
|  | 5c | Give details of treatments received, if relevant. | 5 |
| Outcome | 6a | Clearly define the outcome that is predicted by the prediction model, including how and when assessed. | 6 |
|  | 6b | Report any actions to blind assessment of the outcome to be predicted. | 6 |
| Predictors | 7a | Clearly define all predictors used in developing or validating the multivariable prediction model, including how and when they were measured. | 6-7 |
|  | 7b | Report any actions to blind assessment of predictors for the outcome and other predictors. | 6 |
| Sample size | 8 | Explain how the study size was arrived at. | 7 |
| Missing data | 9 | Describe how missing data were handled (e.g., complete-case analysis, single imputation, multiple imputation) with details of any imputation method. | 7 |
| Statistical analysis methods | 10c | For validation, describe how the predictions were calculated. | 7 |
|  | 10d | Specify all measures used to assess model performance and, if relevant, to compare multiple models. | 7-9 |
|  | 10e | Describe any model updating (e.g., recalibration) arising from the validation, if done. | NA |
| Risk groups | 11 | Provide details on how risk groups were created, if done. | 8 |
| Development vs. validation | 12 | For validation, identify any differences from the development data in setting, eligibility criteria, outcome, and predictors. | 12-13 |
| **Results** | | | |
| Participants | 13a | Describe the flow of participants through the study, including the number of participants with and without the outcome and, if applicable, a summary of the follow-up time. A diagram may be helpful. | 10 Fig1 |
|  | 13b | Describe the characteristics of the participants (basic demographics, clinical features, available predictors), including the number of participants with missing data for predictors and outcome. | 10 Table 1 |
|  | 13c | For validation, show a comparison with the development data of the distribution of important variables (demographics, predictors and outcome). | 12-13 |
| Model performance | 16 | Report performance measures (with CIs) for the prediction model. | 10 |
| Model-updating | 17 | If done, report the results from any model updating (i.e., model specification, model performance). | NA |
| **Discussion** | | | |
| Limitations | 18 | Discuss any limitations of the study (such as nonrepresentative sample, few events per predictor, missing data). | 14 |
| Interpretation | 19a | For validation, discuss the results with reference to performance in the development data, and any other validation data. | 12-13 |
|  | 19b | Give an overall interpretation of the results, considering objectives, limitations, results from similar studies, and other relevant evidence. | 13-14, 15 |
| Implications | 20 | Discuss the potential clinical use of the model and implications for future research. | 14,15 |
| **Other information** | | | |
| Supplementary information | 21 | Provide information about the availability of supplementary resources, such as study protocol, Web calculator, and data sets. | 18 |
| Funding | 22 | Give the source of funding and the role of the funders for the present study. | 1 |

| **Table S2.** Patient demographics by 30-day mortality | | | |  |
| --- | --- | --- | --- | --- |
|  | | | **Total cohort (**n= 17 965) | **High risk cohort**  (n= 1 807) |
| Age at surgery (years), median [IQR] | | | 58 [40-70] | 72 [62-81] |
| Female sex (%) | | | 10 202 (56.8) | 941 (52.1) |
| Surgical urgency (%) | | | | |
|  | | Urgent | 4 211 (23.4) | 696 (38.5) |
|  | | Elective | 13 754 (76.6) | 1 111 (61.5) |
| Surgical complexity (%) | | |  |  |
|  | | Minor | 1 399 (7.8) | - |
|  | | Intermediate | 4 850 (27.0) | - |
|  | | Major | 5 647 (31.4) | 734 (40.6) |
|  | | Xmajor | 2 975 (16.6) | 655 (36.2) |
|  | | Complex | 3 094 (17.2) | 418 (23.1) |
| Malignancy (%) | | | 4 746 (26.4) | 723 (40.0) |
| ASA class (%) | | |  |  |
|  | I | | 5 218 (29.0) | - |
|  | II | | 7 622 (42.4) | - |
|  | III | | 4 486 (25.0) | 1 612 (89.2) |
|  | IV | | 609 (3.4) | 188 (10.4) |
|  | V | | 30 (0.2) | 7 (0.4) |
| Surgical specialty (%) | | |  |  |
|  | | Vascular | 343 (1.9) | - |
|  | | Orthopedic | 4 126 (23.0) | 915 (50.6) |
|  | | Neurosurgery | 1 140 (6.3) | - |
|  | | Obstetrics | 870 (4.8) | 67 (3.7) |
|  | | Urogenital | 2 914 (16.2) | 255 (14.1) |
|  | | Gastrointestinal/abdominal | 2 941 (16.4) | 570 (31.5) |
|  | | Breast | 660 (3.7) | - |
|  | | Thoracic | 614 (1.5) | - |
|  | | Otorhinolaryngologic | 1 291 (7.2) | - |
|  | | Other^a^ | 2 800 (15.6) | - |
|  | | Not classifiable^b^ | 266 (1.5) | - |
| SORT predicted mortality risk | | |  |  |
|  | | Median [IQR], % | 0.3 [0.1-0.8] | 2.4 [1.0-6.3] |
|  | | Mean ± standard deviation, % | 1.2 ± 0.3 | 4.6 ± 0.6 |
| Observed 30-day mortality (%) | | | 284 (1.6) | 102 (5.6) |
| Values are n (%) unless otherwise indicated.  ^a^Including (with percental 30 day mortality): endoscopic (n=623, 4.3%), oral (n=313, 0.03%), endocrine (n=393, 0%), ophthalmologic (n=422, 0.02%), reconstructive (n=675, 1.2%), transplant (n=56, 1.8%), and other surgeries requiring anaesthesia e.g. percutaneous interventions (n=318, 4.1%).  ^b^ No corresponding UK procedure codes were found for these surgical procedures. | | | | |

**
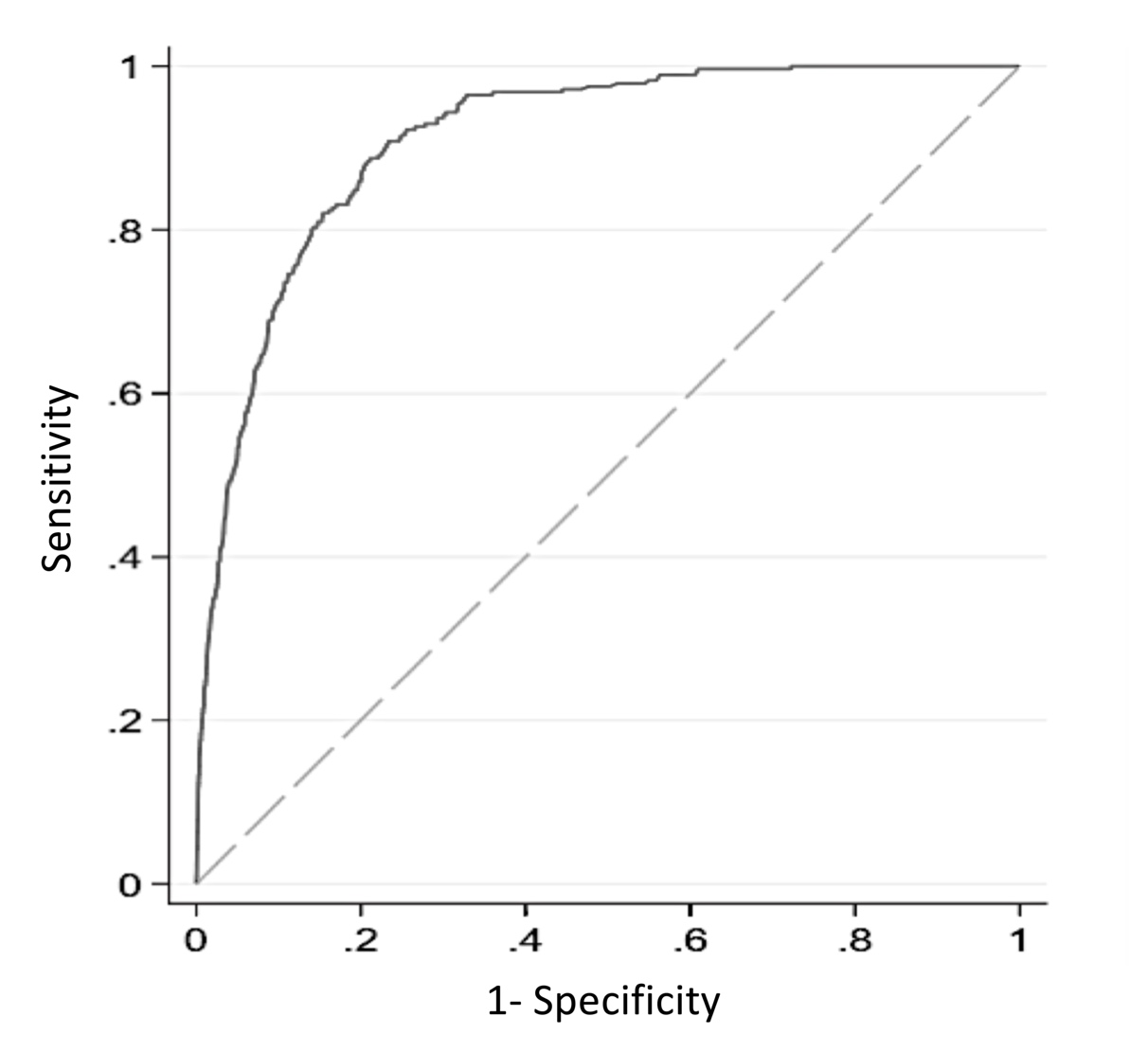
**

Figure S1**. Receiver operating characteristics (ROC) curve.** ROC curve showing discrimination of the re-estimated SORT via 10-fold cross validation for prediction of 30-day mortality. AUROC was 0.91 (95% CI 0.90, 0.93).

The ROC curve of the re-estimated models via 10-fold cross validation is shown in Figure S1. For the sensitivity analysis, high-risk patients were identified using the inclusion criteria for the previously published high-risk cohort^14^: 1)ASA-PS class ≥3; 2) surgical complexity major, complex, or x-major; 3) gastrointestinal, orthopedic, or gynecological/obstetric surgery; 4) age ≥ 18 years old. This resulted in a subgroup of 1807 patients. The demographic data of the high-risk subgroup are presented in Table S2.

**
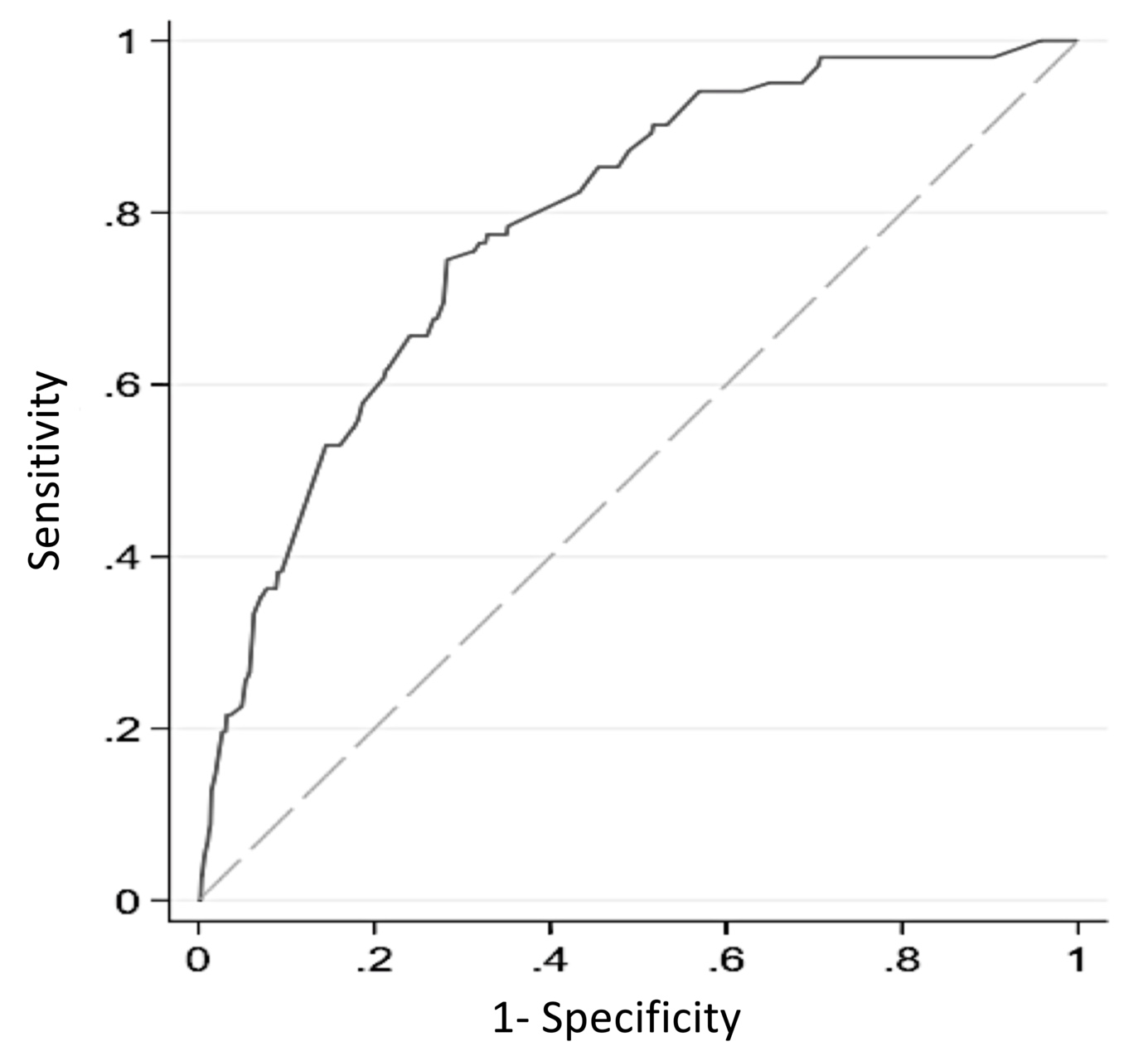
**

**Figure S2. Receiver operating characteristics (ROC) curve.** ROC curve showing discrimination of SORT for prediction of 30-day mortality in the high-risk subgroup (n=1807). AUROC was 0.79 (95% CI 0.74, 0.83).

**
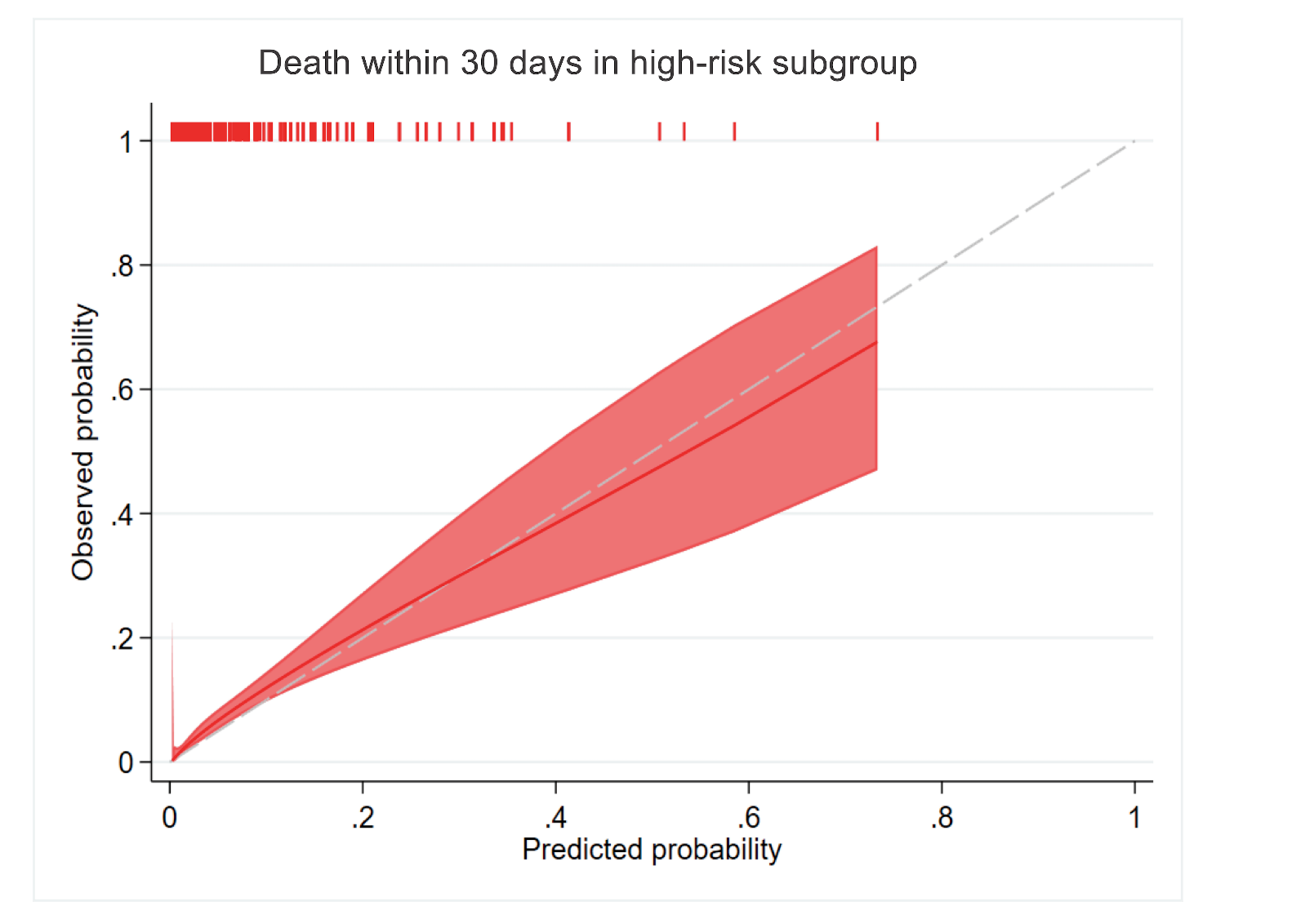
**

**Figure S3. Calibration curve.** The curves depict calibration for mortality at 30 days for the original SORT model in the high-risk subgroup (n=1807). The shaded area around each line shows 95% confidence interval. The slope was 0.89 (95% CI 0.71, 1.08) with an intercept of -0.04 (95% -0.57, 0.49). The dashed line following y=x with an intercept of 0 shows optimal calibration with equal observed vs. predicted mortality.
